# Implication of Epigenetic Alterations of ZEB1 in Colorectal Cancer (CRC) Pathogenesis and Therapy Development

**DOI:** 10.3390/cimb48030276

**Published:** 2026-03-04

**Authors:** Tasnima Kamal, Asma Ul Husna Biswas, Azadur Rahman Bhuiyan, Al-Amin Hossain, Chandan Barai, Yearul Kabir, Farhadul Islam

**Affiliations:** 1 Department of Biochemistry and Molecular Biology, University of Rajshahi, Rajshahi 6205, Bangladesh; rakatasnima2015@gmail.com (T.K.); ontibiswas7@gmail.com (A.U.H.B.); arbhuiyan259@gmail.com (A.R.B.); alaminbmb39@gmail.com (A.-A.H.); chandanbarai64@gmail.com (C.B.); 2 Department of Biochemistry and Molecular Biology, University of Dhaka, Dhaka 1000, Bangladesh; ykabir@yahoo.com; 3 School of Medicine and Dentistry, Gold Coast Campus, Griffith University, Gold Coast 4222, Australia

**Keywords:** colorectal cancer (CRC), ZEB1, epigenetic regulation, epithelial–mesenchymal transition (EMT), histone modification, DNA methylation, cancer progression, therapeutic targets, non-coding RNAs

## Abstract

Colorectal cancer (CRC) is a significant cause of cancer mortality in the world, and its etiology is complicated by genetic and epigenetic changes. As one of the most important tumor progression regulators, Zinc Finger E-box Binding Homeobox 1 (ZEB1) is a transcription factor that has a key role in epithelial–mesenchymal transition (EMT), which is essential in the metastasis, drug resistance, and plasticity of cancer cells in CRC. ZEB1 silences the expression of epithelial markers, including E-cadherin, and it induces the development of mesenchymal properties, such as invasion and metastasis, i.e., tumor aggressiveness. ZEB1 drives epigenetic reprogramming in CRC by coordinating histone deacetylation, histone methylation, and DNA methylation of epithelial tumor suppressor gene promoters and by engaging in reciprocal regulatory interactions with non-coding RNAs, including the miR-200 family. Furthermore, multiple oncogenic signaling cascades, including Wnt/β-catenin, TGF-β, NF-κB, MEK-ERK, JAK/STAT3, and HIF-1α, converge on ZEB1 to amplify its transcriptional and epigenetic activity, positioning ZEB1 as a nodal integrator of extracellular cues and epigenetic reprogramming in CRC metastasis. This review integrates three interconnected regulatory layers, i.e., (1) ZEB1’s direct epigenetic control of target gene expression via histone modification and DNA methylation, (2) post-transcriptional regulation of ZEB1 itself by ncRNAs (miRNAs, circRNAs, and lncRNAs) that create feedback circuits modulating layer 1, and (3) upstream modulation of ZEB1 transcriptional activity by oncogenic signaling pathways (Wnt/β-catenin, TGF-β, NF-κB, MEK-ERK, JAK/STAT3, and HIF-1α) to provide a comprehensive picture of ZEB1 in CRC metastasis and its therapeutic implications.

## 1. Introduction

Colorectal cancer (CRC) is a common and deadly cancer in the world, being the third most frequently diagnosed, and the second most frequent cause of cancer deaths, and more than 1.9 million new cases and more than 930,000 deaths worldwide were estimated in 2020 [1,2]. Although there are advances in screening and multimodal treatment procedures, the prognosis of advanced CRC is still poor because of late diagnosis, metastasis, and resistance to conventional treatment [3]. A growing body of evidence has established that epigenetic dysregulation encompassing aberrant DNA methylation, histone modification, and non-coding RNA activity is a key mechanistic driver of CRC pathogenesis, acting alongside genetic mutations to silence tumor suppressor genes and activate oncogenic programs [4,5]. Among the transcription factors that exploit these epigenetic mechanisms to drive CRC progression, Zinc finger E-box-binding homeobox 1 (ZEB1) has emerged as a central regulator. Epigenetic modifications are heritable changes independent of DNA sequence, including DNA methylation, histone modification, and ncRNA regulation—control gene expression, and contribute to CRC development [6,7,8,9]. These changes can significantly contribute to tumorigenesis by altering the expression of key genes. Among them, ZEB1, also known as TCF8 or DeltaEF1, has emerged as a pivotal transcription factor in CRC development. The two-handed zinc finger protein ZEB1 binds to the E-box (5′-CANNTG-3′) in the immunoglobulin heavy-chain enhancer. It is also known as δEF1, a transcriptional repressor specific to the E-box in the δ-crystallin enhancer core [10].

**Figure 1:**
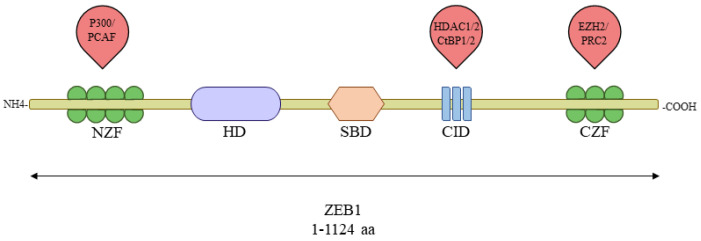
**ZEB1 domain architecture (1124 aa).** Annotation: N-terminal zinc finger cluster (NZF, aa 1–100); homeodomain (HD); Smad-binding domain (SBD); CtBP interaction domain (CID); C-terminal zinc finger cluster (CZF, aa ~1030–1124). Label co-regulator binding at each domain: CID → CtBP1/2–HDAC1/2; CZF → EZH2/PRC2; NZF region → p300/PCAF.

ZEB1, located on human chromosome 10p11.2, has two zinc finger clusters at the N- and C-termini that sandwich a homeodomain, the SBD, and the CtBP interaction domain in the middle (Figure 1). Mass spectrometry revealed shorter ZEB1 transcripts and other transcriptional variations based on non-canonical open reading frames [11]. ZEB1 plays a central role in mediating epithelial–mesenchymal transition (EMT), a process that enables epithelial cells to acquire mesenchymal traits, enhancing their migratory, invasive, and stem-like properties [12,13]. In CRC, overexpression of ZEB1 correlates with poor differentiation, an advanced tumor stage, and reduced overall survival [14]. Mechanistically, ZEB1 represses epithelial markers such as E-cadherin while activating mesenchymal genes, often in cooperation with chromatin-modifying enzymes and signaling pathways like TGF-β and Wnt/β-catenin [15,16]. The impact of ZEB1 goes beyond direct transcriptional repression and also has a major role in regulating the epigenetic landscape of CRC cells. This includes the recruitment of histone-modifying enzymes, the modification of the DNA methylation pattern, and modulating the expression of the microRNA (miRNA) [16]. Even though direct studies of ZEB1 are not described explicitly in the literature, there are a number of studies that clarify the changes in the epigenetics and their role in the events that include silencing of genes and remodeling of chromatin, which are highly similar to the functions identified in ZEB1 in the process of EMT regulation [16,17]. Researchers have determined the epigenetic silencing of the tumor suppressor gene INA by methylation, which highlighted the role of DNA methylation in silencing genes in the course of CRC. ZEB1 has the potential to recruit histone deacetylases (HDACs), such as HDAC1 and HDAC2, to the E-cadherin promoter, resulting in histone deacetylation and transcriptional repression. This mechanism subdues the E-cadherin expression and facilitates EMT [18]. Although the direct connection between ZEB1 and the process of DNA methylation in CRC is still under study, the effect of this protein on EMT may indirectly impact the patterns of methylation by balancing the other epigenetic modifiers [19]. Moreover, miRNAs and especially the miR-200 family can regulate the expression of ZEB1. miR-200 has its site of action on the 3′ untranslated region (UTR) of the ZEB1 mRNA, thereby degrading it or translationally repressing it [20]. According to the latest research, ZEB1 is associated with chemoresistance and immune evasion in CRC by promoting a cancer stem cell (CSC)-like phenotype and regulating the tumor microenvironment. Due to its multifunctional nature, ZEB1 has drawn interest as a prognostic biomarker as well as a possible therapeutic target. Some attempts at inhibiting ZEB1 expression or activity are underway, such as using small-molecule inhibitors, antisense oligonucleotides, and miRNA-based therapies [13,21]. This review hypothesizes that ZEB1 functions as a master epigenetic coordinator in CRC, integrating histone modification, DNA methylation, and ZEB1’s multifaceted roles in CRC metastasis across three interconnected regulatory layers. First, we analyze how ZEB1 functions as a direct epigenetic regulator by recruiting histone-modifying enzymes, modulating DNA methylation patterns, and silencing tumor suppressor and epithelial genes to drive EMT. Second, we examine how ZEB1’s own expression and activity are post-transcriptionally and post-translationally controlled by a network of non-coding RNAs, including miRNAs (particularly the miR-200 family), circRNAs, and lncRNAs, thereby creating feedback circuits that fine-tune ZEB1-mediated epigenetic reprogramming. Third, we discuss how upstream oncogenic signaling pathways, including Wnt/β-catenin, TGF-β, NF-κB, MEK-ERK, JAK/STAT3, and HIF-1α, converge on ZEB1 to amplify its transcriptional and epigenetic activity in CRC. Together, understanding these three layers reveals ZEB1 as a nodal integrator of epigenetic and signaling networks, with significant implications for the development of targeted therapies in CRC. Current reviews on ZEB1 have focused on its role in EMT across various cancers. However, this review uniquely integrates the entire epigenetic network involving ZEB1 in CRC, connecting its epigenetic outputs (histone modification and DNA methylation) with upstream regulation by non-coding RNAs (miRNAs, circRNAs, and lncRNAs) and key oncogenic signaling pathways. Additionally, it evaluates the therapeutic implications of these networks, positioning ZEB1 as a significant target for precision epigenetic therapy in CRC.

### 1.1. ZEB1 as an Epigenetic Regulator in CRC

The direct effect of ZEB1 on the epigenetic landscape of CRC cells is that it regulates chromatin modifiers and creates feedback mechanisms that enhance the expression of ZEB1. ZEB1 is a transcriptional factor that silences the epithelial markers (such as E-cadherin) and promotes mesenchymal markers such as vimentin and N-cadherin [22,23]. ZEB1 binds directly to the promoter of E-cadherin, a typical epithelial protein marker, and suppresses its expression. It identifies the hexamer core (5′-CAGGTG-3′) as a binding site that is identical to the E-box sequence binding site and is a consensus. Interestingly, ZEB1/2 enhances the aggressive nature of cancer, causes the expression of mesenchymal marker proteins, and inhibits the expression of epithelial marker proteins, including E-cadherin. ZEB1 activates the histone methyltransferase of H3K4me3, an active chromatin mark that is enriched at the ZEB1 promoter in EMT-induced cells. ZEB1 recruits co-regulators in a domain-dependent manner (Figure 1): CID engages CtBP1/2–HDAC1/2 for histone deacetylation-mediated epithelial silencing; CZF recruits EZH2/PRC2 for H3K27me3 repression; NZF interacts with p300/PCAF to activate pro-invasive genes (e.g., LAMC2 and uPA) under Wnt/β-catenin conditions. This dual repressor–activator capacity drives ZEB1’s role in CRC. This forms a vicious cycle that continues the high expression of ZEB1 and facilitates aggressive tumority. The clinical importance of this epigenetic network is associated with high co-expression of ZEB1 and SETD1B in patients with CRC, and the high co-expression is associated with a poor prognosis [24]. Epigenetic silencing of epithelial genes through the involvement of ZEB1 also occurs by recruiting chromatin-modifying enzymes to promoters. These are histone deacetylases (HDACs), DNA methyltransferases (DNMTs), and ubiquitin ligases, and they all suppress the expression of genes and induce EMT. It is suggested that interference with the interaction of ZEB1 and the mentioned enzymes could be a possible treatment option to reverse the process of EMT and prevent the progression of the tumor [18]. Therefore, ZEB1 does not act in isolation, but it brings epigenetic modifiers to epigenetically silence the genes that strengthen the EMT phenotype. Table 1 summarizes the functional role of ZEB1 in CRC progression.

### 1.2. Histone Modification

The complicated mechanism of CRC progression is directly linked to epigenetic alterations and is driven by the overexpression and/or inactivation of oncogenes. Histone-modifying enzymes catalyze alterations in histones to regulate gene expression, which is essential in the genesis and progression of CRC [35,36,37,38,39]. Among the transcriptional regulators involved in CRC progression, ZEB1 lacks enzymatic activity; instead, it serves as a transcriptional repressor that recruits the chromatin-modifying complex to occupy promoters. According to recent research studies on CRC, three kinds of modifications are involved in the process of histone modification: acetylation, methylation, and phosphorylation. Not only can the expression patterns of these three histone modifiers act independently, but they can also cross-combine with each other and interact with the DNA methylation mechanism to alter the expression levels of CRC-related genes [29]. ZEB1 potentially regulates five genes that are involved in chromatin modification.

Step 1: Transcriptional Repression—HDAC Recruitment via CtBP

First, ZEB1 also interacts with the C-terminal binding protein (CtBP) and binds histone deacetylases HDAC1 and HDAC2, which also cause deacetylation of histones H3 and H4 at promoters of target genes like CDH1 (E-cadherin), causing transcriptional repression [24].

Step 2: Chromatin Remodeling—H3K27me3 Deposition via EZH2/PRC2

Second, ZEB1 interacts with EZH2 (enhancer of zeste homolog 2), the catalytic subunit of polycomb repressive complex 2 (PRC2), which catalyzes trimethylation of histone H3 at lysine 27 (H3K27me3). This repressive histone suppresses epithelial genes, such as CDH1 and EpCAM [40].

Step 3: Gene Silencing—H3K4 Demethylation via LSD1

Third, LSD1 (KDM1A), the histone demethylase that erases the activating mark H3K4me1/2, is linked to ZEB1 and contributes to transcriptional repression even further [41]. ZEB1 is a molecular beacon that signals LSD1 into the CoREST-CtBP co-repressor complex, causing LSD1 to switch its role of transcriptional activation on genes to repression and allowing dynamic re-programming of epithelial gene expressions during EMT [42]. Lastly, ZEB1 itself interacts with BRG1, which is a chromatin remodeler of the SWI/SNF complex, to directly repress E-cadherin transcription without involving its conventional co-repressor, CtBP. Interfering with the ZEB1-BRG1 interaction restores the expression of E-cadherin and decreases the mesenchymal marker vimentin, which means a transition back to an epithelial phenotype [33]. Therefore, ZEB1 enhances the development of CRC by organizing epigenetic suppression of epithelial genes through the recruitment of HDACs, EZH2, and LSD1. The result of these interactions is chromatin remodeling and EMT, processes that increase tumor invasiveness.

**Figure 2:**
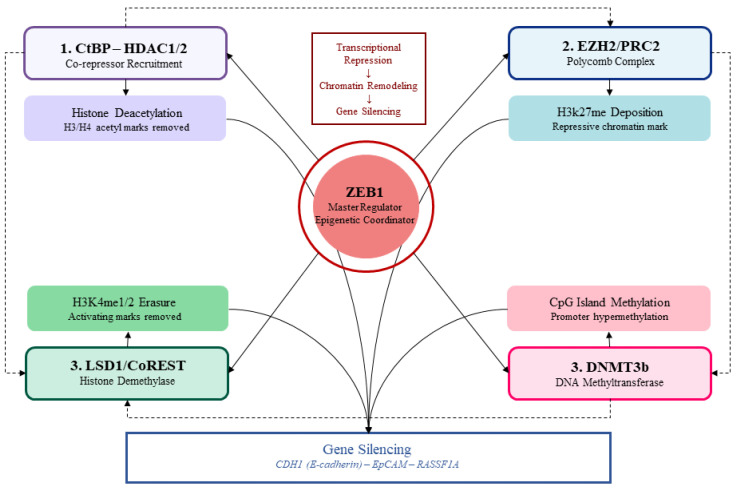
**ZEB1-mediated epigenetic silencing cascade in colorectal cancer.** ZEB1 (the central node) orchestrates stable gene silencing through the sequential recruitment of four co-regulator complexes. Step 1: Via its CtBP interaction domain, ZEB1 recruits CtBP–HDAC1/2 to deacetylate histones H3/H4 at target promoters (transcriptional repression). Step 2: ZEB1 engages EZH2/PRC2 to deposit the repressive H3K27me3 mark (chromatin remodeling). Step 3: ZEB1 recruits LSD1/CoREST to erase the activating H3K4me1/2 marks (gene silencing reinforcement). Step 4: ZEB1 recruits DNMT3b to enforce CpG island hypermethylation (stable epigenetic lock). The convergent output is durable silencing of epithelial and tumor suppressor genes, namely CDH1, EpCAM, and RASSF1A, promoting EMT in CRC. Solid arrows = direct ZEB1-mediated co-regulator recruitment; dashed arrows = sequential cascade flow.

All these sequential steps will result in a multi-layered epigenetic silencing cascade: ZEB1 initially recruits HDACs to remove activating acetyl marks (transcriptional repression), then EZH2/PRC2 to deposit repressive H3K27me3 (chromatin remodeling), and finally coordinates LSD1 to erase the activating H3K4me1/2 marks (stable gene silencing). This sequential activation of epithelial-specific gene promoters to a stable heterochromatin state entraps CR cells in the mesenchymal phenotype (Figure 2).

### 1.3. DNA Methylation

One important epigenetic modification involved in this process is DNA methylation, which adds methyl groups to DNA. While DNA methylation plays a crucial role in normal cellular regulation, it can also contribute to mutagenesis. This alteration is attributed to the alteration in chromatin, which forms an important regulator of gene activation and silencing. Since the development of cancer is usually associated with abnormal expression of genes, changes in the structure of the chromatin, such as changes in aberrant DNA methylation, are commonly seen [43,44,45,46]. In CRC, sporadic and inherited abnormal methylation of cytosine bases in CpG islands in the regulatory gene regions has been frequently reported. Other genes shown to be linked with CRC, such as KRAS, the Rho GTPase family, MACC1, Met, MTA1, and RASSF1A, not only mutate but also show epigenetic changes, especially in the methylation pattern. These genes include RASSF1A, which is directly suppressed by ZEB1 via a methylation-dependent process. ZEB1 is an oncogenic factor that complexes with the RASSF1A promoter along with MUC1-C, which is a major player in cancer biology, and binds the DNA methyltransferase DNMT3b. The result of this recruitment is hypermethylation of CpG islands at the promoter of RASSF1A and transcriptional silencing [47]. The connection between ZEB1 and the DNA methylation machinery is a reciprocal process. MeCP2 (Methyl-CpG binding protein 2) interacts with the transcription factor SPI1 (Spi-1 Proto-Oncogene) and helps in recruiting it to the promoter of ZEB1. After that, SPI1 promotes the expression of ZEB1 at the transcriptional level. ZEB1, in turn, triggers the expression of MMP14, CD133, and SOX2, which keeps CRC in a state of stemness and metastasis [48]. The inter-relationship between epigenetic regulation and the development of CRC is emphasized by this feedback loop.

Methylation-based silencing of epithelial inactivation of metastasis-suppressive genes also occurs via ZEB1. ZEB1 has been reported to silence EpCAM (epithelial cell adhesion molecule) gene expression through the DNA methylation of promoters. The E-box elements in the EpCAM promoter are directly bound by ZEB1 and recruited by epigenetic repressors, i.e., the DNA methyltransferases (DNMTs), and histone modifiers. This promotes hypermethylation of the promoter and silencing of EpCAM transcription during EMT in CRC [24]. EpCAM loss promotes EMT, enhancing cell motility, invasion, and metastatic capacities [49]. Similarly, the methylation status of genes encoding microRNAs (miRNAs) involved in EMT and metastasis, such as miR-9, miR-34, and miR-210, is being investigated as a potential marker for CRC progression. The transcriptional regulation of ZEB1 itself is also subject to epigenetic control. MEF2D directly regulates the transcription of ZEB1 and facilitates histone acetylation at the ZEB1 promoter. MEF2D plays an important role as a central integrator that responds to various tumor microenvironment signals and transduces multiple signals to activate ZEB1 transcription [50]. This regulatory mechanism demonstrates how upstream epigenetic modifications can amplify ZEB1-driven EMT and metastatic programs in CRC. Table 2 lists genes silenced epigenetically by ZEB1.

## 2. Transcriptional Regulation of ZEB1 in CRC

### 2.1. Non-Coding RNA

Non-coding RNAs (ncRNAs), especially microRNAs (miRNAs), regulate biological processes via post-transcriptional gene silencing of mRNA translation [67,68]. Genetic or epigenetic alterations in ncRNA genes disrupt their expression and regulated mechanisms [69,70].

#### 2.1.1. miRNA

In cancer, they act as oncomiRs or tumor suppressors, modulating tumor initiation, progression, and apoptosis [71,72]. In CRC, multiple miRNAs directly or via ceRNA networks negatively regulate ZEB1, influencing metastasis and progression. In CRC, the interplay between miRNAs and ZEB1 represents a critical regulatory network that influences metastatic potential and disease progression. Several miRNAs have been identified as direct negative regulators of ZEB1 expression and function as tumor suppressors in CRC. For example, miR-873-5p directly targets ZEB1, thereby inhibiting cell migration, invasion, and EMT. The miR-873-5p/ZEB1 axis emerges as a potential therapeutic target and prognostic biomarker for CRC, with restoration strategies possibly enhancing anti-metastatic therapies [73]. Similarly, miR-708 is significantly downregulated in CRC tissues and HCT-116 cells, while ZEB1 is markedly upregulated; the two are inversely correlated (*p* < 0.05). miR-708 overexpression decreased phosphorylation of AKT and mTOR (p-AKT, p-mTOR), while ZEB1 overexpression restored these levels, linking the axis to AKT/mTOR pathway inhibition. This pathway drives CRC progression, EMT, and resistance. Thus, miR-708/ZEB1 modulation limits tumor growth and metastasis [74]. Additionally, overexpression of miR-186-5p suppresses proliferation, metastasis, and EMT in cells (LoVo) derived from CRC. The luciferase reporter assay, qRT-PCR, and Western blot analysis demonstrated that miR-186-5p directly targets the 3′UTR of ZEB1 mRNA [75]. Furthermore, YAP1 (Yes Associated Protein 1) expression is elevated in CRC tissues and positively correlates with ZEB1-AS1 levels. YAP1 has potential binding sites with miR-205, which was confirmed by a luciferase assay in CRC cells. Meanwhile, miR-205 was negatively regulated by ZEB1-AS1 in CRC cells. Notably, YAP1 overexpression was able to reverse the inhibitory effects induced by ZEB1-AS1 knockdown, promoting cell proliferation and reducing apoptosis in CRC cells. However, the precise molecular mechanisms underlying YAP1’s role in tumor progression warrant further exploration [76,77]. This indirect regulation through competing endogenous RNA (ceRNA) networks adds another layer of complexity to ZEB1-mediated oncogenic signaling in CRC. The UTRs of Snail and ZEB1 form a competing endogenous RNA (ceRNA) network by competing for binding to miR-34 and miR-200, resulting in synchronized upregulation of both EMT inducers. This ceRNA crosstalk reinforces EMT and therapeutic resistance in CRC by allowing each EMT inducer to indirectly protect the other from miRNA-mediated suppression [78].

#### 2.1.2. circRNA

In CRC, circRNAs frequently function as competing endogenous RNAs (ceRNAs) that sequester miRNAs targeting ZEB1, thereby modulating its expression and influencing tumor progression [79,80]. Several circRNAs have been identified as positive regulators of ZEB1 through miRNA sponging mechanisms. Circ-ZEB1 promotes EMT, a process that enhances the invasive and metastatic potential of CRC cells. The role of circular RNA, namely circWHSC1, has been elucidated in several malignancies [81]. Compared to normal colon epithelial cells, circWHSC1 levels were higher in colon cancer cells. Depletion of circWHSC1 inhibited colon cancer cell viability as well as CRC invasion and migration. Studies indicated that circWHSC1 enhances cell proliferation in CRC both in vitro and in vivo. Findings demonstrated that circWHSC1 targets miR-130a-5p to enhance ZEB1 expression in colon cancer cells by targeting miR-130a-5p/zeb1 signaling in both in vitro and in vivo [82]. Similarly, circ_001860 promotes CRC progression through the miR-582-5p/ZEB1 axis. The upregulation of ZEB1 due to decreased miR-582-5p levels facilitates CRC progression by promoting cell migration, invasion, and metastasis [83]. Additionally, hsa_circ_0001178 sponges multiple microRNAs (miRNAs) that normally inhibit ZEB1 expression, leading to ZEB1 upregulation and promoting invasion and metastasis in CRC [84]. In contrast to oncogenic circRNAs, certain circRNAs function as tumor suppressors by antagonizing ZEB1-mediated oncogenic signaling. For instance, circPTEN1 inhibits the TGF-β/Smad signaling pathway and ZEB1 expression. Specific miRNAs that bind to it and negatively regulate PTEN and the TGF-0 pathway are sponged by it, stimulating the expression of PTEN and tumor-suppressive activities. CircPTEN1 overexpression inhibits cell proliferation, migration, and invasion, which results in the inhibition of cancerous behavior [85]. It shows that the regulation of ZEB1 via circRNA may prove to be counterproductive to CRC progression in the presence of various circRNAs.

#### 2.1.3. LncRNA

In CRC, multiple lncRNAs have been identified as key regulators of ZEB1 expression and activity, predominantly through ceRNA mechanisms that sequester ZEB1-targeting miRNAs [86,87]. Several lncRNAs promote CRC progression by functioning as miRNA sponges, derepressing ZEB1 expression. For example, the lncRNA HCP5 is markedly upregulated in CRC tissues and cell lines, while miR-139-5p is downregulated. Mechanistically, HCP5 functions as a competing endogenous RNA (ceRNA) that sponges miR-139-5p, thereby releasing repression of the transcription factor ZEB1. Rescue experiments further uncover that miR-139-5p inhibition restores ZEB1 amounts and EMT indicators and involves the HCP5/miR-139-5p/ZEB1 axis as a key suppressor of CRC aggressiveness. Additionally, the axis regulates the Wnt/catenin signaling pathway. HCP5 increases catenin activity but is inhibited by miR-139-5p or ZEB1 knockdown, and it is also associated with the axis and wider oncogenic signaling networks [88]. On the same note, the protein expression of the lncRNAs ZFAS1 and ZEB1 is greatly increased in the blood of patients with CRC. miR-200b becomes inhibited by ZFAS1 and does not inhibit ZEB1, which eventually resulting in EMT [89]. The ZFAS1/miR-200b/ZEB1 axis is an emerging diagnostic biomarker with great potential in the diagnosis of CRC since it is highly sensitive and specific. ZFAS1 is an interesting therapeutic target because the targeting of miR-200b sponging would inhibit ZEB1-driven progression [24,64]. Beyond miRNA sponging, lncRNAs also regulate ZEB1 through post-translational mechanisms and protein interactions. For instance, the m6A-induced lncRNA RP11 (RP11-138J23.1) drives CRC cell dissemination by upregulating ZEB1 post-translationally, promoting EMT, migration, invasion, and liver metastasis. Wu et al. found RP11 to be highly expressed in CRC tissues and cells, correlating with advanced stages, as determined by microarray, qRT-PCR, and TCGA data analyses [90]. LINC01413 acts as an oncogenic long non-coding RNA in CRC, promoting cell proliferation and EMT through an hnRNP-K/ZEB1 axis that induces YAP1/TAZ1 nuclear translocation. LINC01413 is upregulated in CRC tissues, as identified via microarray analysis, and correlates with a poor prognosis and advanced stages [91]. In addition to directly regulating ZEB1, some lncRNAs exert oncogenic effects through ZEB1-independent mechanisms while sharing the ZEB1 nomenclature. High expression of the lncRNA ZEB1-AS1 promotes CRC cell proliferation by directly suppressing p15 (CDKN2B), a cell cycle inhibitor, thereby accelerating the G1/S transition and tumor growth. The study by Gong et al. reports ZEB1-AS1 upregulation in CRC tissues and cell lines (e.g., HCT116, SW480), which correlates with a poor prognosis, as shown in the TCGA and patient cohort analysis [92]. This highlights the diverse mechanisms through which the ZEB1 locus and its associated regulatory RNAs contribute to CRC pathogenesis.

### 2.2. Pathways Involving ZEB1 in CRC Pathogenesis

**Figure 3:**
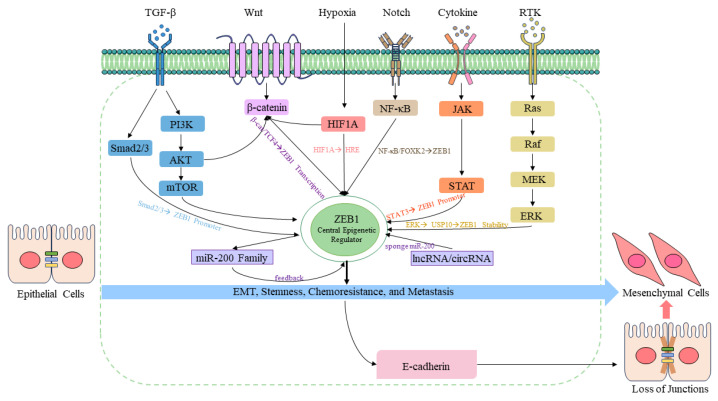
**Oncogenic signaling pathways converging on ZEB1 to drive epithelial–mesenchymal transition (EMT) in colorectal cancer.** Major extracellular stimuli, such as TGF-β, Wnt, hypoxia, Notch, cytokines, and receptor tyrosine kinase (RTK) ligands, activate intracellular signaling cascades that directly regulate ZEB1 transcription or protein stability. TGF-β induces SMAD2/3, which binds the *ZEB1* promoter to activate transcription; Wnt signaling liberates β-catenin, which forms a β-catenin/TCF4 complex that directly drives *ZEB1* expression; HIF-1α binds hypoxia-response elements (HREs) in the *ZEB1* promoter under low-oxygen conditions; NF-κB acts through the FOXK2 transcription factor to transactivate *ZEB1*; JAK-activated STAT3 directly binds and activates the *ZEB1* promoter; and the MEK-ERK cascade stabilizes the ZEB1 protein post-translationally via USP10 phosphorylation. ZEB1 (central red node) integrates all these inputs as a master epigenetic transcriptional regulator. A self-reinforcing feedback loop is established through the mutual repression between ZEB1 and the miR-200 family: ZEB1 suppresses miR-200 expression (blunt-ended line), while lncRNAs and circRNAs sponge miR-200, further amplifying ZEB1 activity. Elevated ZEB1 expression represses E-cadherin (blunt-ended line), driving loss of epithelial junctions and channels downstream of EMT outputs; resulting in stemness, chemoresistance, and metastasis; and culminating in the acquisition of a mesenchymal phenotype. Solid arrows = direct activation; blunt ends = inhibition/repression.

EMT is driven by multiple signaling pathways. The following sections analyze how key oncogenic signaling cascades, including Wnt/β-catenin, NF-κB, TGF-β, MEK-ERK, HIF-1α, and JAK/STAT3, converge directly on ZEB1 to drive CRC metastasis (Figure 3).

### 2.3. Wnt/β-Catenin Signaling Pathway

ZEB1 is directly and indirectly regulated by Wnt/β-catenin signaling, which is usually constitutively active in CRC due to APC or β-catenin mutations (direct transcriptional activator; synergizes with NF-κB and MEK-ERK). Nuclear 2-catenin binds the TCF/LEF transcription factors to induce the transcription of Wnt target genes, one of them being ZEB1 [93,94], upon activation.

It is recorded that the β-catenin/TCF4 complex is directly involved in the direct induction of ZEB1 transcription, which leads to EMT and enhanced cell motility. This is achieved by the recruitment of the PRC2 complex that trimethylates H3K27 to silence tumor-suppressive microRNAs in the CRC cells [32,95]. In line with this, Spaderna et al. (2008) established that Wnt signaling stimulation increased ZEB1 expression, which, in effect, repressed E-cadherin and led to the further invasion of CRC cells [14]. Furthermore, the ZEB1-AS1/miR-181a-5p/ZEB1 axis amplifies this regulation. ZEB1-AS1 functions as a competing endogenous RNA, directly sponging miR-181a-5p (as confirmed by luciferase and RIP assays). This interaction de-represses ZEB1, leading to activation of the Wnt/β-catenin signaling pathway (increased β-catenin, TCF4, Cyclin D1, Myc, and MMP-7 expression) and downstream EMT effectors (Snail and vimentin) while reducing E-cadherin expression. miR-181a-5p inhibition partially restores the effects of ZEB1-AS1 knockdown, confirming that the ZEB1-AS1/miR-181a-5p/ZEB1 axis drives CRC proliferation, migration, and invasion [96].

Beyond transcriptional regulation, ZEB1 physically interacts with β-catenin/TCF4, co-activating pro-metastatic genes, including LAMC2 (cell adhesion/migration) and uPA (urokinase plasminogen activator), and extracellular matrix degradation/invasion and converting ZEB1 into a transcriptional activator [29,97,98]. ZEB1 and TCF4 not only co-bind to the same DNA regulatory regions but also modulate each other’s activity, depending on the presence of co-factors like p300 (activator) or CtBP/BRG1 (repressors). This dual interaction adds another layer of regulatory complexity. ZEB1 also interacts with chromatin modifiers like BRG1 and HDACs, enhancing the transcription of Wnt target genes and contributing to EMT and stemness traits [20,99]. In addition, Wnt/β-catenin signaling requires ZEB1 for optimal uPA induction. ZEB1 has been reported to enhance the invasiveness of CRC in two aspects through enhancement or down-regulation of uPA and the plasminogen activator inhibitor-1 (PAI-1) mRNA and protein in CRC cells. ZEB1 directly interacts with the uPA promoter and triggers transcription via the recruitment of the co-activator p300 [30]. This interaction succeeds in transforming ZEB1 into a transcriptional activator, which facilitates invasive and metastatic behavior in CRC. This axis is regulated by a number of upstream factors: DDX3 stimulates β-catenin/ZEB1 through the Akt/GSK-3b pathway in APC cells or KRAS-mutated cells [61]. In addition, Wnt signaling is activated by RHBDD1, inducing phosphorylated 0-catenin that enhances EMT and stemness by upregulating ZEB1 [100].

### 2.4. NF-κB Signaling Pathway

ZEB1 is a master regulator of EMT, metastasis, and therapy resistance in CRC [101] by interacting with NF-κB signaling, serving as a central hub that integrates multiple oncogenic pathways (indirect activator via FOXK2; synergizes with Wnt/β-catenin and TGF-β). Activation of NF-κB drives ZEB1 expression and promotes EMT and invasion [102]. NF-κB activation by the EGFR/ERK pathway is a significant one. EGFR/ERK stimulation favors the activation of NF-kB, which interacts with the promoter region of the FOXK2 transcription factor that transactivates ZEB1. This EGFR/ERK/NF-κB/FOXK2 cascade mediates epithelial repression of E-cadherin by ZEB1 as well as epithelial invasion and metastasis [103].

NF-κB activity augmentation through phosphorylation of p65 induces an inflammation-induced increase in IL-8 secretion and a pro-inflammatory tumor microenvironment, permitting EMT, angiogenesis, and metastasis with the help of ZEB1 activation [104]. Although there is no direct evidence that IL-8 triggers ZEB1 initiation in CRC, RNF183-stimulated NF-κB IL-8 signaling can activate EMT, in part, by upregulating ZEB1. NF-κB signaling combines with other oncogenic pathways to control ZEB1 expression. PSMD4 is stimulated by cytoplasmic Nrf2 (cNrf2), leading to the activation of NF-κB and PI3K/AKT signaling—with convergent effects on the β-catenin/TCF pathway—and resulting in direct binding of the ZEB1 promoter and the subsequent promoter of ZEB1 transcription. This cNrf2-NF-κB/β-catenin/TCF pathway enhances the migration and invasion of the cells in CRC through ZEB1 [15,105], establishing a control loop that entraps cells in a mesenchymal state. Notably, there are also negative regulators of NF-κB that aid EMT via ZEB1. Loss of ASPP1 inhibits NF-κB activity, promoting Snail2 expression, another EMT transcription factor that cooperates with ZEB1 to stabilize EMT. Snail2 further suppresses the miR-200 family, which normally inhibits ZEB1, thereby amplifying ZEB1 activity and sustaining EMT progression [106,107]. Collectively, these data highlighted that the diverse upstream signals, including EGFR/ERK, ncRNA–SP1, RNF183/IL-8, cNrf2/PSMD4, and ASPP1/Snail2, result in NF-κB-mediated regulation of ZEB1, positioning ZEB1 as a master driver of EMT and metastatic plasticity in CRC progression.

### 2.5. TGF-β Signaling Pathway

TGF-β signaling serves different functions at different stages of cancer progression. In early tumor lesions, TGF-β acts as a tumor suppressor by inhibiting cell growth, inducing cell cycle arrest, and promoting apoptosis (direct SMAD-mediated activator; synergizes with KRAS/ERK and Wnt) [108,109]. Accordingly, this signaling activation suppresses the proliferation of colon epithelial cells in early-stage CRC [110]. However, with tumor progression, this pathway becomes a pro-metastatic driver promoting immune suppression, neo-angiogenesis, and metastasis [111,112,113]. ZEB1, along with other transcription factors, is involved in this functional shift in TGF-β signaling [114,115]. In the canonical pathway, ligand binding to type II TGF-β receptors (TGFBR2) recruits and activates type I TGF-β receptors (TGFBR1), which phosphorylate SMAD2/3. These receptors oligomerize with SMAD4 and translocate to the nucleus, where the SMAD complex binds the ZEB1 promoter to initiate its transcription [114]. Thus, in CRC, constitutive SMAD7 upregulation uncouples receptor turnover from signal attenuation, blunting TGF-β’s tumor-suppressive effects while allowing persistent SMAD2/3 activity to drive ZEB1 induction [116,117,118].

Additionally, in CRC, miR-200 genes are silenced through promoter hypermethylation and repressive histone marks [119], while the ZEB1 protein binds E-boxes in these gene regions to maintain their repression [120,121]. This sustains high ZEB1 levels, enhancing cancer cell mobility and invasiveness. Also, beyond canonical SMAD signaling, mutations in TGF-β pathway components can activate non-canonical pathways that further drive ZEB1 expression. A deletion mutation of three alanine residues in the TGFBR1 signal peptide, as detected in CRC, reduces SMAD2/3 phosphorylation while enhancing p38 and ERK1/2 MAP kinase activation [122]. Furthermore, SMAD4 mutations cause loss of SMAD4 function, leading to continued MAPK pathway activation. Continuous MAPK-ERK signaling via these mutations directly upregulates ZEB1 transcription. Moreover, oncogenic KRAS mutations (Q22K and G12D) lock KRAS in its active GTP-bound form, synergizing with TGF-β to amplify ERK1/2 phosphorylation and consequently upregulating ZEB1, thereby driving partial EMT in CRC models [123,124]. Once induced, the ZEB1 protein represses miR-200, which typically suppresses ZEB1 by binding to its mRNA [125]. Newly synthesized ZEB1 proteins interact with SMAD3 and stabilize SMAD3–SMAD4 binding at the ZEB1 promoter, creating a self-reinforcing feedback loop that sustains ZEB1 expression [126]. Findings suggest that DAPK1 downregulation activates ZEB1, promoting EMT and CSC stemness via the WNT and TGF-β pathways. However, since ZEB1 knockdown in sh-DAPK1 cells did not reduce TGF-β1 expression, ZEB1 likely functions downstream of DAPK1 and TGF-β rather than within a feedback loop, though the detailed mechanism requires further clarification [62]. Conversely, Grainyhead-like-2 (GRHL2), a member of the mammalian Grainyhead family of wound-healing regulatory transcription factors, suppresses EMT and restores anoikis sensitivity through at least two mechanisms: direct repression of ZEB1 expression and inhibition of TGF-β signaling [123]. Once ZEB1 is activated by this pathway, it drives EMT by downregulating crucial epithelial markers like E-cadherin (enabling cell detachment) [58,127], ZO-3 (disassembly of tight junctions) [128], the miR-200 family [123] while upregulating mesenchymal markers, such as vimentin (cytoskeletal reorganization) [34], fibronectin (extracellular matrix remodeling), and N-cadherin (mesenchymal cell–cell contacts). This EMT reduces apoptosis, increases cell migration and invasion, and decreases sensitivity to oxaliplatin (OXA) in colon cancer cells [129].

### 2.6. MEK-ERK Pathway Regulation of ZEB1 in CRC

The course of CRC is largely driven by MEK-ERK signaling, which controls ZEB1-mediated EMT, stemness, chemoresistance, and metastasis (post-translational stabilizer; amplified by KRAS and EGFR mutations). This pathway is modulated by various upstream regulators, including circular RNAs, long non-coding RNAs, kinases, phosphatases, and proteases, which converge to regulate ZEB1 at multiple levels: transcriptional activation, post-translational stabilization, and signal amplitude control [130].

The MEK-ERK pathway is activated by a number of factors to promote ZEB1-mediated oncogenic programs. To illustrate this case, the circular RNA circRAPGEF5, which is a molecular controller, sustains downstream signaling to stabilize ZEB1 and provoke its transcriptional activity to encourage EMT and metastasis but prevents hyperactivation-induced apoptosis in KRAS-mutant CRC cells [15,65]. In this way, the rhomboid family protease RHBDD1, which is overexpressed in CRC, is able to cleave pro-TGF α in the absence of the metalloprotein ADAM. The secreted TGF 2 interacts with EGFR and activates the RAF-MEK-ERK pathway, which induces CRCC cell proliferation and metastasis [131]. Moreover, lncRNA SLCO4A1-AS1 is an oncogenic controller as it stimulates EGFR/MAPK signaling, thus stimulating the MEK-ERK cascade and stimulating the growth, migration, and progression of the tumor in the form of CRC [132]. Once activated, the MEK-ERK pathway is amplified by kinases that enhance ZEB1 function. Polo-like kinase 1 (PLK1) phosphorylates components of the CRAF–MEK1/2–ERK1/2 cascade, linking cell cycle control to EMT, tumor aggressiveness, cancer stem cell characteristics, and chemoresistance in CRC [133].

ZEB1 is also stabilized by the MEK-ERK pathway via a post-translational mechanism. Under normal cell conditions, USP10 removes ZEB1 K27-ubiquitinated ubiquitin to allow K48-mediated ubiquitination and proteasomal degradation. But in CRC cells, MEK-ERK phosphorylates USP10 at Ser236, maintains K27-linked ubiquitination, inhibits K48-linked degradation, and stabilizes ZEB1, enhancing migration and metastasis [123,134]. Under normal conditions, negative regulators restrain MEK-ERK-driven ZEB1 activity. PHLPP (PH domain and leucine-rich repeat protein phosphatase) suppresses tumors by dephosphorylating RAF1 and maintaining low ZEB1 activity. Its downregulation in CRC hyperactivates RAF-MEK-ERK signaling, stabilizes ZEB1, and promotes migration, invasion, and tumor progression [135]. The loss of such negative regulators in CRC, thus, contributes to sustained MEK-ERK-ZEB1 signaling and aggressive tumor behavior. Together, these regulators show a complex network that governs transcriptional, post-translational, and signaling-level modulation of MEK–MEK-ERK-mediated ZEB1 activity. This network ultimately contributes to EMT, stemness, chemoresistance, and metastasis in CRC.

### 2.7. HIF-1α and ZEB1 in CRC Pathogenesis

HIF-1α (hypoxia-inducible factor 1-alpha) is a transcription factor that plays a key role in the cellular response to low-oxygen (hypoxia) conditions (hypoxia-driven transcriptional activator; converges with TGF-β at the invasive front) [136]. In CRC, hypoxia creates a selective pressure that promotes aggressive tumor phenotypes, with HIF-1α serving as a critical mediator of ZEB1-driven EMT. HIF-1α binds to hypoxia response elements (HREs) in the ZEB1 promoter, leading to increased ZEB1 transcription under hypoxia. Both HIF-1α and ZEB1 are positively associated with vimentin, an important mesenchymal marker of EMT, whereas they are negatively associated with E-cadherin expression. While HIF-1α and ZEB1 are both widely considered as tumor-initiating factors, ZEB1 functions as a direct downstream target of HIF-1α, suggesting a novel molecular mechanism for HIF-1α-induced EMT and cancer metastasis [18]. Consistent with this mechanism, ZEB1 is expressed in human CRC samples, particularly in dedifferentiated tumor cells at the invasive front [20].

Other than HIF-1α, another HIF family member (HIF3alpha1) is involved in the metastasis mediated by ZEB1, but via a different mechanism. Hypoxia-inducible factor 3alpha1 (HIF-3alpha1) enhances CRC to liver metastasis through EMT and augmentation of iron intake by means of upregulating ZEB1 and the transferrin receptor (TFRC). These results demonstrate the HIF-3 area1/ZEB1/TFRC axis as a promising therapeutic option in CRC liver metastasis, which supplements the already recognized ZEB1-regulated EMT regulators such as miR-873-5p and LINC01413. EMT inhibitors can be more effective after targeting HIF-3 alpha 1 or iron chelators [137]. This observation indicates that ZEB1 activation through hypoxia not only encourages the EMT process but also regulates the metabolism of cancer cells to favor metastatic colonization.

### 2.8. JAK/STAT3-Mediated Regulation of ZEB1 in CRC

Activation of STAT3 leads to an increase in ZEB1 expression and directs EMT development by fixing the ZEB1 expression in CRC (direct promoter-binding activator; independent of SMAD signaling). STAT3 has a direct interaction with the promoter of ZEB1 and controls ZEB1 transcription activity [58]. Research has demonstrated that depending on the cell knockout status of STAT3, ZEB1 prevents EMT caused by STAT3, which indicates that ZEB1 is a downstream target of STAT3 in the invasion and silencing of E-cadherin. Accordingly, ZEB1 could be involved in the cell invasion and downregulation of E-cadherin that was caused by STAT3 in CRC cells. In addition to STAT3, another STAT family member, helps in the regulation of ZEB1 via an upstream transcriptional cascade. E2F1, one of the important transcription factors in the progression of CRC, has been reported to facilitate the migration and invasion of cells in CR. Notably, STAT6 protein levels correlated with E2F1 expression across multiple CRC cell lines. Mechanistic studies revealed that SP3, a member of the SP transcription factor family, directly regulates STAT6 transcription, and its upregulation appears to depend on E2F1. These findings highlight the E2F1/SP3/STAT6 axis as a potential regulatory pathway that contributes to STAT6 signaling in CRC [138]. The expression of STATA6 is triggered by cytokine signaling to induce EMT via ZEB1. The signaling is initiated by interleukin-13 (IL-13), which is bound by IL-13R alpha 1, which is highly expressed in the majority of CRC cell lines. The complex of receptors attracts JAK kinases, which phosphorylate STAT6 and activate the canonical pathway of JAK/STAT6. After that, STAT6 moves into the nucleus when activated and binds to the promoter of ZEB1, thus triggering transcription of ZEB1. High levels of ZEB1 promote EMT by regulating the levels of EMT-related markers [138,139]. In addition, IL-4R, comprising both the IL-4R alpha and IL-13R alpha1 subunits, can be activated by IL-13, though the affinity of IL-4 is stronger. It has been demonstrated by previous research that IL-4 and IL-13 have overlapping signaling pathways, especially the activation of STAT6 by the JAK/STAT pathway in CRC cells. ZEB1 is an important IL-4/STAT6 downstream effector that supports its presence in the regulation of EMT in the progression of CRC [138,139,140]. The interplay between IL-4 and IL-13 signaling on the induction of ZEB1 via STAT6 is an important observation that is associated with the role of cytokine-mediated inflammatory mechanisms in enhancing CRC metastasis (Figure 3).

Collectively, the above-discussed signaling pathways (Figure 3) control ZEB1 independently and synergistically, placing ZEB1 in the center of nodal integration of oncogenic signals in CRC. All pathways interact with ZEB1 using different molecular mediators: Wnt/3-catenin interacts directly with 3-catenin/TCF4 to induce transcriptional activation; TGF-b interacts with SMAD2/3, binding the ZEB1 promoter; NF-κB activates ZEB1 with the help of the FOXK2 transcriptional cascade; MEK-ERK stabilizes ZEB1 post-translationally with the help of USP10 phosphorylation; JAK/STAT3 binds the ZEB1 promoter directly. More importantly, these signaling pathways do not act in isolation; they interact and enhance one another: KRAS mutations cooperate with TGF-1 to enhance ERK1/2-initiated ZEB1 upregulation; Wnt/3-catenin and NF intersect with TGF-1 to enhance ZEB1 induction at the invasive front. Upon activation, ZEB1 strengthens all these cues in an epigenetic manner by silencing miR-200 family members, which would otherwise silence several pathway components, forming a self-sustaining oncogenic feed-forward loop. This integrative position of ZEB1 renders it a highly appealing and attractive therapeutic target.

### 2.9. ZEB1 in Tumor Microenvironment and Immune Modulation

The central role of ZEB1 in mediating tumor progression in the microenvironment of the CRC is through a mechanism that combines the signals of stromal cells, hypoxia, cytokines, and extracellular matrix components. To restore the tumor microenvironment, ZEB1 promotes tumor invasion by increasing cancer cell motility by disrupting intercellular adhesion, consequently promoting migration. Microenvironmental stimuli in CRC activate transcriptional activity of myocyte enhancer factor 2D (MEF2D) to activate ZEB1, promoting EMT and metastasis [50,141,142,143]. Myofibroblastic characteristics and inhibition of inflammatory activation are facilitated by the expression of ZEB1 in cancer-associated fibroblasts (CAFs), which suppress anti-tumor immunity and promote metastasis. Fibroblast-specific Zeb1 deficiency positively affects CAF barrier function, but it enhances cytokine signatures and lymphocyte recruitment, which makes tumors susceptible to immune checkpoint therapies, highlighting ZEB1 as a potential immunotherapeutic target and immune-prognostic biomarker in the tumor microenvironment [144].

In addition to the classical EMT sensors, namely E-cadherin and vimentin, ZEB1 has been demonstrated to co-purify with cancer stem cell (CSC) conductors in CRC, yet again indicating its contribution towards aggressive maintenance of tumors. It is important to note that ZEB1 expression is correlated with high concentration levels of ALDH (aldehyde dehydrogenase), which is a working marker of CRC stem cells [145,146]. There is higher ZEB1 expression and mesenchymal and chemotherapy resistance in ALDH-high CRC cell populations, indicating that the ZEB1-ALDH axis identifies an especially aggressive CSC subpopulation [147]. Secondly, ZEB1 was also found to co-express with other mesenchymal and stemness markers such as CD44, CD133, and fibronectin at invasive tumors of CRC, and patterns of co-expression were associated with a high TNM stage, lymph node metastasis, and low overall survival [148]. These links show that in addition to promoting EMT, ZEB1 maintains the stem-like state that facilitates metastatic colonization and therapeutic evasion in CRC.

Beyond tumor cells, ZEB1 in cancer-associated fibroblasts (CAFs) influences the tumor microenvironment by promoting myofibroblastic features, suppressing anti-tumor immunity, and facilitating metastasis. Loss of ZEB1 in CAFs increases immune cell infiltration and enhances immunotherapy sensitivity, suggesting a dual role in tumor progression and immune regulation [143]. ZEB1 links EMT to immunotherapy resistance through three mechanisms: it induces PD-L1 expression in invading cancer cells, directly inhibits T-cell activation, and upregulates CD47, driving M2 polarization of tumor-associated macrophages (TAMs). This ZEB1-mediated reprogramming of the tumor microenvironment shields invading cancer cells from immune surveillance and contributes to resistance against immune checkpoint blockade therapies. Reciprocal repression between GRHL2 and ZEB1, each regulated by microenvironmental factors, further modulates this process. GRHL2 represses ZEB1 by inhibiting at least three of its activators, demonstrating a regulatory checkpoint that can restrain ZEB1-driven EMT in response to specific microenvironmental cues [123,148]. One of the main characteristics of the tumor microenvironment is hypoxia. The ZEB1 promoter’s hypoxia-response regions are directly bound by HIF-1α, which increases transcription of the gene. In the hypoxic TME, this overexpression enhances invasion and metastasis, increases cancer cell motility, and promotes EMT [149,150]. Hypoxia further regulates non-coding RNAs that modulate ZEB1. For example, the long non-coding RNA HOTAIR is upregulated under hypoxic conditions and suppresses miR-1277-5p, leading to increased ZEB1 expression, which drives EMT and enhances invasion and metastatic potential [151,152,153,154,155,156]. IL-1β induces EMT in CRC cells by upregulating ZEB1, and silencing ZEB1 reverses IL-1β-induced EMT along with stem cell characteristics [156]. Cytokines from the tumor microenvironment, including IL-1β, promote chronic inflammation, which in turn enhances the characteristics of cancer stem cells, such as the formation of spheres and the overexpression of stemness markers like Bmi1 and Nestin. Additionally, IL-1β promotes ZEB1 expression, creating a feedback loop that suppresses the miR-200 family, a typical Bmi1 suppressor, maintaining cancer stemness and bolstering EMT [135,157,158,159]. Extracellular matrix components are also involved: Collagen I, reduced upon OSBPL2 downregulation, enhances focal adhesion and migration. OSBPL2 depletion promotes these effects via the VCAN/AREG/EREG–ERK signaling axis and a PARP1–ZEB1-mediated pathway, further contributing to EMT and invasion in CRC cells [160]. Lastly, hypoxic CAFs generate exosomes with lower levels of miR-200b-3p, thereby making CRC cells more invasive. By suppressing miR-200b-3p in CAFs, ZEB1 and E2F3 are upregulated in recipient tumor cells, which connect stromal hypoxia to EMT and tumor growth [161,162]. These pathways collectively demonstrate how ZEB1 integrates diverse signals from the tumor microenvironment, including hypoxia, inflammatory cytokines, extracellular matrix remodeling, immune checkpoint activation, and stromal tumor communication, to promote invasion, immune evasion, stemness, and EMT in CRC.

## 3. Therapeutic Implication of ZEB1 in CRC

### 3.1. ZEB1-Mediated Therapy and Drug Resistance in CRC

CRC continues to be one of the major causes of cancer mortality worldwide [160]. Despite all improvements in cancer treatment strategies, more than 90% of metastatic CRC-related deaths are due to multidrug resistance [163]. Therefore, to develop new precision medicines to overcome current therapeutic failures in CRC, the factors and their fundamental mechanisms mediating therapy resistance need to be clarified. Chemoresistance is often associated with metastases [162]. ZEB1 can influence cellular processes involved in EMT, drug efflux [163], DNA damage repair [162], and cancer stem cell maintenance [163] to establish therapy resistance in various cancers. ZEB1 contributes to chemoresistance and radioresistance in CRC by promoting EMT and enhancing DNA damage response pathways. Its upregulation is linked to a poor response to chemotherapy and increased cancer stem cell-like properties [103,164,165]. Targeting the ZEB1 network, including its epigenetic and signaling partners, may help overcome resistance to current therapies.

### 3.2. ZEB1-Mediated DNA Damage Response and Repair

One of the primary mechanisms through which ZEB1 confers chemoresistance is by enhancing DNA damage response and repairing pathways. ZEB1 causes 5-fluorouracil (5-FU) resistance in CRC by repairing 5-FU-induced DNA damage via upregulation of key DNA damage response factors such as NBS1, RNF8, and RNF168. 5-FU treatment of ZEB1 knockdown cells (SW480KD and RKOKD) showed increased DNA damage and chromosome abnormalities with higher γ-H2AX foci, extended DNA tails, and decreased homologous recombination (HR) repair efficiency. Conversely, overexpression of ZEB1 reduced DNA damage, increased chromosome stability, and restored HR repair after 5-FU treatment [164]. Stem-like cells exhibit enhanced DNA damage response (DDR) and DNA repair capacity, as well as self-renewal, or chemoresistance. In contrast, functional experiments have shown that ZEB1 induces chemoresistance regardless of whether other EMT-related changes occur. ZEB1 has also been identified as an important regulator of DDR by the formation of a ZEB1/p300/PCAF complex and direct interaction with the ATM kinase, which has been linked to radioresistance [162,163]. It was shown to promote therapy resistance through EMT-dependent and -independent mechanisms, in which ZEB1 contributes to DNA repair by interacting with USP7 to stabilize CHK1 [103,164]. Interestingly, DNA repair genes (USP17, CHD1L, and DUX4), which can promote drug resistance, were identified as downstream targets of ZEB1. Knockdown of ZEB1 in the LoVo cell line resulted in upregulation of USP17, CHD1L, and DUX4 and increased sensitivity to cisplatin and etoposide, with enhanced cell cycle arrest and apoptosis [13]. Stem-like cells manifest enhanced DNA damage response and repair capacity, self-renewal, and chemoresistance. Functional experiments have demonstrated that ZEB1 induces chemoresistance regardless of whether other EMT-related changes occur, underscoring its direct role in DNA repair mechanisms [166].

### 3.3. ZEB1 and Resistance to Chemotherapeutic Agents and Inhibitors

ZEB1 contributes to resistance against multiple chemotherapeutic agents commonly used in CRC treatment. When doxorubicin-resistant DLD1 cells (DLD1 DR) were analyzed, higher expression of ZEB1 was found, which positively correlated with TCF4 expression. ZEB1 knockdown in DLD1 DR cells resulted in reduced doxorubicin resistance. Furthermore, overexpression of ZEB1 in normal doxorubicin-sensitive DLD1 cell lines increased doxorubicin resistance, demonstrating ZEB1’s role in mediating resistance [166]. EMT is significantly implicated in oxaliplatin (OXA) resistance, with ZEB1 playing a pivotal role in EMT-mediated OXA sensitivity in colon cancer cells [129]. ZEB1 knockdown in the OXA-resistant HCT116/OXA cell line restored oxaliplatin sensitivity in the resistant cells with reduced cell proliferation, invasion, migration, and reversal of the EMT phenotype and increased apoptosis [167]. It was revealed that the HOTAIR/miR-1277-5p/ZEB1 axis is involved in hypoxia-mediated oxaliplatin resistance in the LoVo, HT-29, and HCT116 cell lines. ZEB1 knockdown in these cells under hypoxic conditions showed more sensitivity to oxaliplatin, reduced proliferation, and reversed EMT marker changes [168].

**Figure 4:**
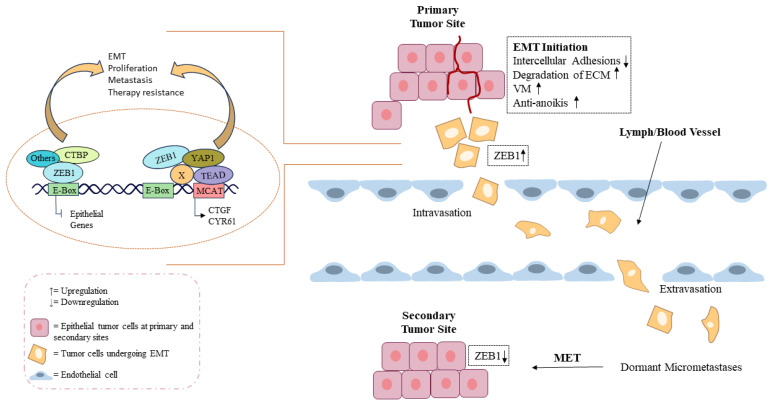
**ZEB1 as a master transcriptional regulator of epithelial–mesenchymal transition, tumor cell dissemination, and metastatic colonization in colorectal cancer.** The figure illustrates the dual role of ZEB1 as a transcriptional regulator and metastatic driver in colorectal cancer (CRC), integrating molecular mechanisms with the sequential steps of tumor dissemination. Left inset (molecular mechanism): ZEB1 functions at two distinct E-box elements in target gene promoters. At epithelial gene promoters (left E-box), ZEB1 recruits the co-repressor CtBP and associated chromatin-modifying complexes (labeled “Others”) to silence epithelial gene programs, including E-cadherin (CDH1). At a second E-box (right), ZEB1 interacts with YAP1, the transcriptional co-activator TEAD, and the MCAT element-binding factor (labeled “X”) to activate the pro-invasive target genes CTGF and CYR61. The resulting gene expression program drives EMT, proliferation, metastasis, and therapy resistance (curved arrows, top). Primary tumor site (top center): EMT initiation at the primary tumor is characterized by reduced intercellular adhesions, degradation of the extracellular matrix (ECM), acquisition of vascular mimicry (VM), and upregulation of anti-anoikis mechanisms. These events coincide with ZEB1 upregulation (ZEB1 ↑) in tumor cells at the invasive front, triggering intravasation into lymphatic or blood vessels. Dissemination (right panels): ZEB1-high mesenchymal tumor cells (shown in yellow/orange) intravasate into the vasculature (upper right), circulate, and subsequently extravasate at distant organ sites (lower right), where they may initially form dormant micrometastases. Secondary tumor site (bottom center): At the secondary site, downregulation of ZEB1 (ZEB1 ↓) permits mesenchymal-to-epithelial transition (MET), enabling dormant micrometastases to re-establish epithelial colonies and form overt secondary tumors. Together, the figure positions ZEB1 as the central molecular switch coordinating epigenetic repression of epithelial identity, activation of pro-invasive transcriptional programs, and the dynamic regulation of EMT–MET plasticity that underlies CRC metastatic colonization. Solid arrows = direct activation; blunt ends = inhibition/repression.

ZEB1 also mediates resistance to histone deacetylase (HDAC) inhibitors. HCT-R, a butyrate-resistant p300-deficient HCT-116 cell line with cross-resistance to other HDAC inhibitors like vorinostat and LBH589, showed significant ZEB1 upregulation that is promoted by CBP-mediated Wnt signaling, and this overexpression was found to be responsible for drug resistance [164,169]. Knockdown of ZEB1 rendered HCT-R cells more sensitive to HDAC inhibitors, with reversal of EMT characteristics. When ZEB1 was overexpressed in butyrate-sensitive, p300-expressing HCT-116 cells, they also showed higher drug resistance and an EMT phenotype [170]. Non-coding RNAs further modulate ZEB1-mediated drug resistance. Knockdown of circ-ZEB1 in SW480 and RKO CRC cells showed reduced oxaliplatin and 5-FU resistance with an increased level of miR-200c. Researchers demonstrated that circ-ZEB1 acts as a sponge for miR-200c, disrupting its normal function in preventing drug resistance [65]. This highlights the complex regulatory networks involving ZEB1 that contribute to chemoresistance in CRC. Several upstream factors regulate ZEB1 expression to promote chemoresistance. It has been reported that cNrf-2-induced upregulation of PSMD4 promoted aggressive cells through the NF-κB/AKT/β-catenin/ZEB1 cascade, suggesting that PSMD4 expression may promote tumor aggressiveness and chemoresistance in colorectal cancer [154]. Similarly, downregulation of death-associated protein kinase 1 (DAPK1) may facilitate metastasis and chemoresistance in cancer cells via several mechanisms. Downregulation of DAPK1 is closely associated with chemoresistance and metastasis in CRC by promoting the stemness of CSCs and the EMT process [62]. Figure 4 illustrates the ZEB1/2-driven EMT-MET cycle, highlighting initiation at primary colorectal tumors, intravasation through lymphatic and blood vessels, and MET-mediated secondary tumor formation with therapy resistance (Figure 4).

### 3.4. Drug Design and Development Targeting ZEB1 in CRC

ZEB1 represents a promising therapeutic target in CRC due to its central role in promoting EMT, metastasis, chemoresistance, and immune evasion. ZEB facilitates the epigenetic silencing of E-cadherin by recruiting multiple chromatin enzymes of the E-cadherin promoter, such as histone deacetylases (HDACs), DNA methyltransferase (DNMT), and ubiquitin ligase. Destruction of the connection between ZEB1 and these chromatin-modifying enzymes may represent an efficient way for treating cancer. In BRAF-mutant CRCs, ZEB1 paradoxically inhibits EMT and is associated with a better prognosis, highlighting the importance of the tumor’s genetic background when considering ZEB1 as a therapeutic target [24,83,162].

#### 3.4.1. Targeting Upstream Regulators of ZEB1

Several upstream signaling pathways that activate ZEB1 have emerged as therapeutic targets. Targeting STAT3, which promotes CRC progression by inducing EMT; enhancing invasive and clonogenic potential; and conferring resistance to chemotherapeutic agents such as fluorouracil and etoposide may be a promising strategy to overcome EMT-associated chemoresistance in CRC [57,171]. The neurotransmitter receptor HTR2B, which is highly expressed in CRC tissues and associated with a poor prognosis, activates ZEB1 transcription through the Akt/mTOR/S6K1/CREB1 signaling cascade. Activated CREB1 binds to the ZEB1 promoter, driving EMT and metastasis, while HTR2B-specific antagonist (RS127445) treatment significantly ameliorates metastasis and reverses EMT in mouse models. This identifies the HTR2B-CREB1-ZEB1 axis as a potential therapeutic target and establishes HTR2B as a prognostic biomarker in CRC [172,173]. Additionally, ursolic acid (UA) inhibited the viability of HCT-116 and HCT-8 colon cancer cells in a dose- and time-dependent manner. UA markedly downregulated key mediators of the TGF-β1 signaling cascade: TGF-β1, phosphorylated Smad2/3, phosphorylated FAK, and the transcription factor ZEB1. Concomitantly, UA upregulated the miR-200 family, with miR-200c exhibiting the greatest increase. The resulting repression of ZEB1 lowered the mesenchymal marker N-cadherin while leaving the epithelial marker E-cadherin unchanged. Its ability to target the TGF-β1/ZEB1/miR-200c network positions it as a potential therapeutic agent for CRC [18]. DAPK1–ZEB1 may lie at the interface of the TGF-β and WNT pathways and participate in both CSC and EMT processes. The DAPK1–ZEB1 pathway is a potential therapeutic target for inhibition of chemoresistance and metastasis [62].

#### 3.4.2. Targeting Non-Coding RNAs in the ZEB1 Network

MicroRNAs that directly target ZEB1 offer promising therapeutic avenues. For instance, miRNA-186-5p influences the proliferation, metastasis, and EMT of colon cancer cells by regulating ZEB1. Therefore, it may be a promising therapeutic target for CRC [77,174,175,176]. miR-708 is a novel therapeutic agent that can suppress CRC progression by targeting ZEB1 and attenuating AKT/mTOR signaling [71]. The HCP5/miR-139-5p/ZEB1/Wnt cascade has been identified as a novel therapeutic target for CRC, offering a mechanistic bridge between lncRNA dysregulation and EMT-driven metastasis. Silencing HCP5 or overexpressing miR-139-5p suppresses cell viability, migratory, and invasive capacities, whereas miR-139-5p inhibition reverses these effects and restores ZEB1 expression [174]. Beyond microRNAs, long non-coding RNAs and circular RNAs that regulate ZEB1 represent viable therapeutic targets. Targeting ZEB1-AS1 (e.g., siRNA or antisense approaches) suppresses tumor growth and reverses EMT-driven phenotypes, indicating that ZEB1-AS1 is a promising therapeutic target for CRC intervention [106,175]. It has been identified that the circular RNA circWHSC1 promotes colon cancer cell proliferation by modulating the miR-130a-5p/zeb1 signaling pathway both in vitro and in vivo. Thus, circWHSC1 can be a novel target in CRC pathogenesis and in elucidating potential processes, offering new insights into CRC development and the exploration of effective treatments [84]. The m6A/RP11/hnRNPA2B1/Siah1-Fbxo45/ZEB1 axis positions RP11 as a CRC-specific biomarker and therapeutic target to block metastasis. Targeting m6A or RP11 could disrupt ZEB1-driven EMT, complementing prior miRNA/ZEB1 studies in a conversation context [92].

Moreover, the CAFs’ expression of ZEB1 contributes to the tumor microenvironment, which affects inflammation and the immunotherapy response [172,173]. Therefore, CAFs can be a viable drug design and development tool. This multi-compartmental targeting strategy, which targets ZEB1 on tumor cells and stromal cells, could be more beneficial therapeutically. Together, combined targeting strategies that targeted ZEB1 and its regulatory network (e.g., Wnt, STAT3, and epigenetic modifiers) can reverse EMT, decrease metastasis, and overcome drug resistance [162].

### 3.5. Challenges and Potential Risks of Epigenetic Therapies Targeting ZEB1

While epigenetic therapies targeting ZEB1 hold significant promise for CRC treatment, several critical challenges must be addressed before clinical translation. The first weakness is that it is not target-specific. Specifically, HDAC and DNMT inhibitors influence genome-wide histone acetylation and epigenetic patterns of DNA methylation and cause off-target phenotypes such as activation of oncogenic pathways or suppression of tumor suppressors in normal tissues [64,165]. These drugs have systemic toxicity, such as myelosuppression, gastrointestinal effects, and fatigue effects, which tend to induce dose-limiting toxicity that can impair therapeutic efficacy [163,164]. Another serious challenge is tumor heterogeneity. CRC has a high level of intra-tumoral heterogeneity in terms of different levels of ZEB1 in cell subpopulations [26,27]. Low-ZEB1-expression cells can be resistant to ZEB1-based therapies and are used as reservoirs to cause recurrence of the disease. In addition, blocking ZEB1 can induce stimulation of other EMT transcription factors, including SNAIL or TWIST, to preserve the mesenchymal phenotype despite the blocking of ZEB1 [80,163]. The reversibility of the epigenetic changes indicates that the effects of the therapeutic approach can be short-lived, and cells resume the repressive chromatin patterns after the withdrawal of the therapeutic intervention [66].

The therapeutic strategies are even complicated by the context-dependent role of ZEB1. The inhibition of EMT by ZEB1 is paradoxical, and the inhibitor is also associated with improved prognosis in BRAF-mutant CRCs, which indicates that ZEB1 inhibition can play contradictory roles depending on the genetic background of tumors [24,83,128,164]. The clinical implementation will be difficult without reliable biomarkers that can be used to stratify the patients. Also, the therapeutic RNA as an miR-200 mimic is associated with serious delivery challenges, including rapid degradation, the lack of cellular uptake, and the inability to penetrate tumors [21,75,77]. Further efforts to overcome these issues should concentrate on creating more discriminating ZEB1 degraders, tumor-targeted delivery, and combination methodologies to tailor many EMT pathways and extensive molecular profiling to determine which patients are most likely to respond to ZEB1-directed therapies [162,175,176,177].

## 4. Conclusions

CRC has been a major cause of cancer-related deaths in the world, with metastasis and resistance to therapy being the main factors that lead to poor patient outcomes. This review critically analyzes the epigenetic regulatory systems by which ZEB1 coordinates CRC progression and specifically its contribution to EMT, chromatin remodeling, and promoting aggressive tumor phenotypes. As the evidence presented in this paper shows, ZEB1 is not only a transcriptional repressor but also a key epigenetic coordinator of CRC. Recruitment of chromatin-modifying complexes, such as histone deacetylases (HDAC1/2), the polycomb repressive complex protein EZH2, and the histone demethylase LSD1-ZEB1, creates stable silencing of epithelial identity genes, including CDH1 and EpCAM. At the same time, ZEB1 interacts with DNA methyltransferases to enforce tumor suppressor promoter hypermethylation (with RASSF1A) to provide a compounded epigenetic barrier to their re-expression. An example of self-reinforcing epigenetic loops that sustain elevated ZEB1 expression in aggressive cells of CRC is the transcriptional activation of the histone methyltransferase SETD1B by ZEB1, which subsequently enhances the presence of an activating mark, H3K4me3, that itself is at the ZEB1 promoter.

Contextualizing ZEB1 within the broader EMT transcription factor landscape reveals additional regulatory layers. SNAI2 (Slug) promotes cancer progression by upregulating NADSYN1, which interacts with PHB to maintain mitochondrial integrity and survival [177]. Given ZEB1-SNI2 co-regulation of overlapping gene sets and mesenchymal maintenance in CRC, ZEB1 may similarly drive metabolic reprogramming (e.g., via NADSYN1–PHB axes), offering new therapeutic targets.

One significant theme that comes up in the literature is a mutual regulatory interaction between ZEB1 and non-coding RNAs, specifically the miR-200 family. A bistable switch mechanism is generated by this double-negative feedback loop, where ZEB1 suppresses the expression of miR-200s, and the members of the miR-200 family regulate the ZEB1 mRNA, which can be used to lock cells in either state, namely the epithelial or mesenchymal state. Other strata of non-coding RNA control, such as long non-coding RNAs (HCP5, ZEB1-AS1, and LINC01021) and circular RNAs (circWHSC1 and circ-ZEB1), also complicate the regulation of ZEB1 and outline the complexity of the post-transcriptional networks controlling EMT in CRC.

The partaking of ZEB1 in various oncogenic signal transduction projects, such as the Wnt/3-catenin, TGF- -bit, NF- -bit, JAK/STAT, and MAPK cascades, places it in a nodal position to pay attention to various microenvironmental cues that mediate coordinated transcriptional responses. Of particular interest is the ability of ZEB1 to be context-dependent, since in the majority of instances, it acts as a transcriptional repressor via co-repression with CtBP, but activation of the Wnt signal can induce a shift to a transcriptional activator by displacing CtBP/BRG1 and recruiting the p300 acetyltransferase. This combinatory plasticity enables ZEB1 to induce pro-metastatic genes like LAMC2 and uPA under particular signaling conditions, providing a mechanistic insight of how EMT programs may block epithelial genes and trigger invasion-promoting genes at the same time.

Clinically, the existing evidence indicates a strong association of ZEB1 in mediating resistance to conventional chemotherapeutic and targeted therapies via the amplification of DNA damage response pathways, the acquisition of cancer stem cell phenotypes, and tumor microenvironment remodeling (5-fluorouracil, oxaliplatin, and doxorubicin). This mechanistic basis of this chemoresistance seems to have multifactorial components (EMT-dependent pathways, including altered drug uptake/efflux and enhanced survival signal) and EMT-independent pathways (direct interaction with DNA repair machinery and USP7-mediated stabilization of CHK1). Nevertheless, some significant caveats and context-dependencies have already appeared based on the literature under review. The observation that ZEB1 is redundant in the EMT of LS174T colon cancer cells when SNAIL1 is still adequately expressed highlights the fact that there are other EMT-regulatory circuits and cautions against simplistic models. Moreover, ZEB1 also appears to suppress EMT and is associated with improved prognosis in BRAF-mutant CRCs, indicating the critical role of the oncogenic context in determining ZEB1 activity. The observations highlight that the development of therapeutic plans based on ZEB1 must consider the tumor genetic background and the specific combination of active oncogenic pathways.

## 5. Limitations and Future Perspective

There are critical gaps in our knowledge that should be explored further. To begin, although it is known that ZEB1 recruits chromatin-modifying factors to the promoters of genes, it has been demonstrated that the chromatin occupancy of ZEB1 in CRC is genome-wide. The dynamics of occupancy patterns of chromatin-modifying factors at ZEB1 promoters during EMT plasticity are not fully described. Second, the comparable roles of ZEB1 and ZEB2 in the progression of the disease in CRC, as well as the degree of functional redundancy or specialization of these paralogs, should be systematically tested. Third, post-translational modifications of ZEB1, such as phosphorylation, ubiquitination, and acetylation, and their role in the stability, nuclear localization, and transcriptional activity of ZEB1 should be further studied mechanistically. Fourth, ZEB1 expression in stromal cells (especially cancer-associated fibroblasts) and its role in tumor–immune interactions and immunotherapy are new fields of study that need to be evaluated further.

Therapeutically, several intervention approaches that would regulate ZEB1 and its regulatory networks have preclinical potential. These involve restoring the expression of the miR-200 family using miRNA mimics or demethylating reagents; inhibiting upstream transcriptional activators, including STAT3, MEF2D, or β-catenin/TCF4; disrupting ZEB1–chromatin modifier interactions; targeting regulatory long non-coding RNAs; and identifying natural compounds that suppress ZEB1 by pathway modulation. Nevertheless, to translate these strategies into clinical value, selective bioavailable agents will have to be developed, and the predictive biomarkers have to be identified to select the patients who are most likely to respond to ZEB1-directed therapies.

Overall, ZEB1 can be considered a clinical target due to its role as a global epigenetic controller that coordinates a wide range of signaling pathways to produce epigenetic reprogramming that leads to EMT, stemness, metastasis, and drug resistance. The high-priority therapeutic targets of ZEB1 and its regulatory network are supported by its central role in cancer biology, as well as excellent prognostic relationships and mechanistic connections to clinical failure in the context of treatment. Such studies combining genomics, epigenomics, and single-cell technology will be necessary in the future to obtain a full understanding of the context-dependent activity of ZEB1, develop vulnerabilities that can be used in therapies, and finally apply this mechanistic understanding to obtain better clinical outcomes in patients with colorectal cancer. With the field moving to precision oncology, integrating ZEB1 status and its upstream regulators into clinical decision-making algorithms could make it possible to support more effective and personalized treatment plans that result in better survival and quality of life for patients with this difficult disease.

## Figures and Tables

**Table 1:** Functional role of ZEB1 in colorectal cancer.

Cell Lines and Experimental Models	Function of ZEB1	References
CRC and pancreatic cancer cells	Represses stemness-inhibiting microRNAs	[20]
SW480 and SW620 CRC cells	Inhibits senescence	[25]
SW480 and HCT-116	Promotes metastasis and loss of cell polarity	[14]
LS174T CRC cells	Not required for EMT	[26]
Human CRC sample analyses	Regulates miR-200c in EMT	[27]
HCT-116 CRC cells	ZEB1-hTERT complex inhibits E-cadherin expression	[28]
SW480, SW620, and HCT-116 CRC cells	ZEB1 and TCF4 reciprocally modulate each other’s transcriptional activity	[29]
SW480, HCT116, and Colo320 CRC cells	Regulates the plasminogen proteolytic system by inducing uPA and inhibiting PAI-1	[30]
HCT-116 CRC cells	Promotes vasculogenic mimicry through EMT induction	[31]
HCT-116 and SW480 CRC cells	Promotes metastasis and loss of cell polarity	[32]
SW480 CRC cells	Represses E-cadherin expression and induces EMT	[14]
HCT-116 CRC cells	Promotes EMT	[33]
HCT-116 and SW480 CRC cells	Promotes tumor invasiveness	[34]

**Table 2:** ZEB1-associated epigenetically silenced genes in CRC and their functional classification.

Function	Gene	ZEB1 Connection	References
Wnt Signaling/Tumor Suppressors	*APC*	Wnt pathway regulator; loss activates β-catenin, leading to ZEB1 upregulation.	[51]
	*AXIN2*	Negative regulator of Wnt; methylation sustains Wnt signaling and ZEB1 activation.	[51]
	*HLTF*	Silencing promotes genomic instability and EMT; associated with ZEB1-high tumors.	[52]
	*RASSF1A*	Tumor suppressor; its silencing facilitates EMT and may correlate with ZEB1 expression.	[52,53]
	*RASSF2A*	Same as above, especially in the Wnt-EMT context.	[54]
	*RUNX1*	Tumor suppressor possibly repressed by ZEB1; loss promotes metastasis.	[55]
Transcription/Chromatin Regulation	*GATA4*	Differentiation factor; methylation supports ZEB1-driven EMT.	[56]
	*GATA6*	Same as above.	[56]
	*MSX1*	Developmental gene; associated with EMT and stemness features enhanced by ZEB1.	[57]
	*JARID2*	Same as above.	[57]
Cell Adhesion Molecules	*CDH1 (E-cadherin)*	Canonical ZEB1 repression target—ZEB1 binding silences CDH1 to induce EMT.	[58]
	*CDH13 (H-cadherin)*	Possible ZEB1 target; cadherins often repressed during EMT.	[17]
	*ITGA4*	ZEB1 may regulate integrins to promote migration/invasion during EMT.	[29]
	*RECK*	MMP inhibitor; indirectly repressed by ZEB1 to increase invasion.	[59]
	*SIX1*	CDH1 repression and EMT in CRC cells were correlated with post-transcriptional ZEB1 activation and miR-200-family transcriptional repression.	[60]
Stemness/Developmental Markers	*MSX1*	(Repeat) ZEB1 linked to EMT + stemness features.	[61]
	CD133	CSC marker; expression enriched in ZEB1-high CRC tumors.	[62]
Metabolism/DNA Repair/Epigenetics	*WNT5A*	Non-canonical Wnt ligand; activated by ZEB1 during EMT.	[63]
	*RAR-β*	Loss promotes EMT; ZEB1 may repress RA signaling.	[64]
Receptors/Neural Function	Vimentin	Classic ZEB1-induced EMT marker; hallmark of mesenchymal state.	[59,65]
Others/Miscellaneous	*SPARC*	Positively regulated by ZEB1; promotes EMT, invasion, and metastasis.	[66]

## Data Availability

No new data were created or analyzed in this study. Data sharing does not apply to this article.

## Abbreviations

AKT	Protein kinase B.
APC	Adenomatous polyposis coli.
ASPP1	Apoptosis-stimulating of p53 protein 1.
ATM	Ataxia telangiectasia mutated.
BRG1	Brahma-related gene 1.
CAF	Cancer-associated fibroblast.
CDH1	Cadherin 1 (E-cadherin).
ceRNA	Competing endogenous RNA.
CHK1	Checkpoint kinase 1.
circRNA	Circular RNA.
CRC	Colorectal cancer.
CREB1	cAMP-responsive element-binding protein 1.
CSC	Cancer stem cell.
CtBP	C-terminal binding protein.
DAPK1	Death-associated protein kinase 1.
DDR	DNA damage response.
DNMT	DNA methyltransferase.
E2F1	E2F transcription factor 1.
ECM	Extracellular matrix.
EGFR	Epidermal growth factor receptor.
EMT	Epithelial–mesenchymal transition.
EpCAM	Epithelial cell adhesion molecule.
ERK	Extracellular signal-regulated kinase.
EZH2	Enhancer of zeste homolog 2.
FOXK2	Forkhead box K2.
GRHL2	Grainyhead-like-2.
GSK-3β	Glycogen synthase kinase-3 beta.
H3K4me3	Histone H3 lysine 4 trimethylation.
H3K27me3	Histone H3 lysine 27 trimethylation.
HAT	Histone acetyltransferase.
HDAC	Histone deacetylase.
HIF-1α	Hypoxia-inducible factor 1-alpha.
HIF-3α1	Hypoxia-inducible factor 3-alpha 1.
hnRNP-K	Heterogeneous nuclear ribonucleoprotein K.
HR	Homologous recombination.
HRE	Hypoxia response element.
hTERT	Human telomerase reverse transcriptase.
HTR2B	5-hydroxytryptamine receptor 2B.
IL	Interleukin.
JAK	Janus kinase.
KRAS	Kirsten rat sarcoma viral oncogene.
LAMC2	Laminin subunit gamma-2.
lncRNA	Long non-coding RNA.
LOXL2	Lysyl oxidase-like 2.
LSD1	Lysine-specific demethylase 1 (KDM1A).
m6A	N6-methyladenosine.
MAPK	Mitogen-activated protein kinase.
MeCP2	Methyl-CpG binding protein 2.
MEF2D	Myocyte enhancer factor 2D.
MEK	Mitogen-activated protein kinase kinase.
MET	Mesenchymal–epithelial transition.
miRNA	MicroRNA.
MMP	Matrix metalloproteinase.
mTOR	Mammalian target of rapamycin.
ncRNA	Non-coding RNA.
NF-κB	Nuclear factor kappa B.
Nrf2	Nuclear factor erythroid 2-related factor 2.
OXA	Oxaliplatin.
p-AKT	Phosphorylated AKT.
p-mTOR	Phosphorylated mTOR.
PAI-1	Plasminogen activator inhibitor-1.
PARP1	Poly (ADP-ribose) polymerase 1.
PCAF	P300/CBP-associated factor.
PHLPP	PH domain and leucine-rich repeat protein phosphatase.
PI3K	Phosphoinositide 3-kinase.
PLK1	Polo-like kinase 1.
PRC2	Polycomb repressive complex 2.
PSMD4	Proteasome 26S subunit non-ATPase 4.
PTEN	Phosphatase and tensin homolog.
qRT-PCR	Quantitative reverse transcription polymerase chain reaction.
RAF	Rapidly accelerated fibrosarcoma.
RAS	Rat sarcoma.
RASSF1A	Ras association domain family member 1A.
RHBDD1	Rhomboid domain containing 1.
RIP	RNA immunoprecipitation.
RNF183	Ring finger protein 183.
S6K1	Ribosomal protein S6 kinase B1.
SETD1B	SET domain containing 1B.
SMAD	Mothers against decapentaplegic homolog.
SP1	Specificity protein 1.
SP3	Specificity protein 3.
SPI1	Spi-1 proto-oncogene.
STAT	Signal transducer and activator of transcription.
SWI/SNF	SWItch/Sucrose Non-Fermentable.
TAM	Tumor-associated macrophage.
TCGA	The Cancer Genome Atlas.
TCF	T-cell factor.
TCF4	Transcription factor 4.
TGF-β	Transforming growth factor-beta.
TGFBR	Transforming growth factor-beta receptor.
TFRC	Transferrin receptor.
TMB	Tumor mutation burden.
TME	Tumor microenvironment.
TNM	Tumor, node, metastasis.
TP53	Tumor protein p53.
uPA	Urokinase plasminogen activator.
USP	Ubiquitin-specific protease.
UTR	Untranslated region.
YAP1	Yes-associated protein 1.
ZEB1	Zinc finger E-box binding homeobox 1.
ZEB1-AS1	ZEB1 antisense RNA 1.
ZNF248	Zinc finger protein 248.
5-FU	5-fluorouracil.
